# Evaluation of Antibiotic-Releasing Triphasic Bone Void Filler In-Vitro

**DOI:** 10.3390/jfb9040055

**Published:** 2018-09-21

**Authors:** Michael Harris, Hamza Ahmed, Leslie Pace, Jon Minter, Michael Neel, Jessica Jennings

**Affiliations:** 1 Department of Biomedical Engineering, University of Memphis, Memphis, TN 38152, USA; mhrris18@memphis.edu (M.H.); hahmed@memphis.edu (H.A.); lrpace@memphis.edu; (L.P.); 2 Northside Hospital System, Atlanta, GA 30342, USA; Jon.MinterM.D@northside.com; 3 Division of Orthopaedics, St. Jude Children’s Research Hospital, Memphis, TN 38105, USA; MNeel@mskgroup.org

**Keywords:** bone void filler, calcium composites, infection

## Abstract

Bone void fillers (BVFs) containing calcium sulfate, tricalcium phosphate (TCP), and hydroxyapatite can be loaded with antibiotics for infection treatment or prevention under surgeon-directed use. The aim of this study was to characterize the handling and elution properties of a triphasic BVF loaded with common antibiotics. BVF was mixed with vancomycin and/or tobramycin to form pellets, and the set time was recorded. A partial refreshment elution study was conducted with time points at 4, 8, and 24 h, as well as 2, 7, 14, 28, and 42 days. Effects on dissolution were evaluated in a 14-day dissolution study. Set time increased to over 1 h for groups containing tobramycin, although vancomycin had a minimal effect. Pellets continued to elute antibiotics throughout the 42-day elution study, suggesting efficacy for the treatment or prevention of orthopedic infections. BVF containing vancomycin or tobramycin showed similar dissolution at 14 days compared to BVF without antibiotics; however, BVF containing both antibiotics showed significantly more dissolution.

## 1. Introduction

Bone grafting is a well-established surgical practice in which osseous defects created by trauma, tumor resection, or arthroplasty are filled using donor-harvested bone or by using other synthetic alternatives [1,2,3,4,5,6,7]. Autografts, sections of bone harvested from the patient’s iliac crest, are considered the gold standard of bone grafts due to their osteoconductive, osteoinductive, and osteogenic properties. These procedures, however, require a second surgical site and have limitations including pain, complications of the harvest site, and limited amounts of bone [2,4,5,8,9,10,11]. Allografts are sometimes harvested from corpses or hip prosthesis procedures and used to fill boney defects; however, these suffer from risk of disease transmission and immune-rejection, and typically lose osteogenic properties during processing [9]. Synthetic, calcium-based cements and ceramics have been available for several decades as an alternative to allografts and autografts. Three common types of calcium bone void fillers (BVF) used clinically are calcium sulfate, tricalcium phosphate, and hydroxyapatite. These synthetic BVFs serve as osteoconductive scaffolds and may exhibit osteoinductive properties depending on the macro and micro porosity of the scaffold [7,12]. Unlike calcium sulfate-based BVFs, tricalcium phosphate (TCP) and hydroxyapatite (HA) are stable at physiologic pH and must be degraded by osteoclasts or macrophages [13]. These BVFs therefore require a degree of interconnected porosity to allow vascularization and osteoblast/osteoclast infiltration to achieve rapid integration with the surrounding bone tissue [10,14]. This can be difficult to achieve in BVFs mixed within the operating room; however, porosity can be increased by adding calcium sulfate that will naturally resorb upon implantation, creating channels in the TCP or hydroxyapatite for cellular ingrowth [13,15]. Combination BVFs have appeared on the market containing mixtures of calcium sulfate, TCP, and/or hydroxyapatite to achieve optimum dissolution rates, porosity, and mechanical properties. 

It is well-documented that local antibiotic delivery with polymethylmethacrylate (PMMA) greatly reduces the number musculoskeletal surgical site infections and improves clearance of musculoskeletal infections when used adjunctively with systemic antibiotics [16,17]. PMMA does have some disadvantages compared to other systems, such as the need for a second surgery to remove temporary drug delivery implants and continued low-level antibiotic elution from permanent implants that can drive the development of antibiotic-resistant bacteria. Calcium BVFs can be easily loaded with antibiotics by mixing the drugs into the calcium phosphate/sulfate powder or the liquid to form a biodegradable antibiotic delivery system, eliminating the need for follow-up surgery and reducing concerns about antibiotic resistance [18]. Antibiotics are commonly added for the treatment of active infections, such as hip arthroplasties or osteomyelitis, or for infection prophylaxis in the case of traumatic injury. Calcium-based BVFs are often prepared under surgeon direction at the time of surgery using antibiotics available in the operating room. Prior work has shown that antibiotics can affect the dissolution and precipitation-hardening reactions of calcium phosphates, thereby increasing set time at a rate dependent on antibiotic concentration within the BVF [19]. Each antibiotic/BVF combination must therefore be evaluated to ensure that the BVF set time does not unnecessarily prolong surgery. 

Triphasic BVF offers potential advantages in mechanical properties and dissolution rates compared to traditional BVF. However, there is currently a lack of relevant data regarding the handling, elution, and dissolution properties of triphasic mixtures when combined with antibiotics. In surgeon-directed use of BVF for antibiotic delivery and bone regeneration, the most commonly used antibiotics are vancomycin and tobramycin. Powdered antibiotic for reconstitution may be added to BVF powder during the mixing process prior to casting into pellets for packing into the defect. Therefore, the objectives of this study were to determine whether the addition of powdered antibiotic at clinically available quantities affects the handling, elution, or dissolution properties of triphasic BVF beads containing calcium sulfate, hydroxyapatite, and TCP. 

## 2. Results

### 2.1. Set Time

Using friability as a guide, an orthopedic surgeon declared the set time for BVF without antibiotics as 7 min. This increased slightly to 8.5 min with the addition of 1 g vancomycin to the BVF powder. BVF containing tobramycin or a combination of tobramycin and vancomycin did not set within the 1.5 h study period; however, they were declared set after sitting overnight for a total of 15 h. Vicat testing, according to ASTM C472, showed that BVF set at 3.5 min without antibiotics and 5 min with vancomycin only. BVF with tobramycin required 21 h to set in accordance with the standard; however, BVF with both vancomycin and tobramycin set within 3 h. 

### 2.2. Elution

**Figure 1:**
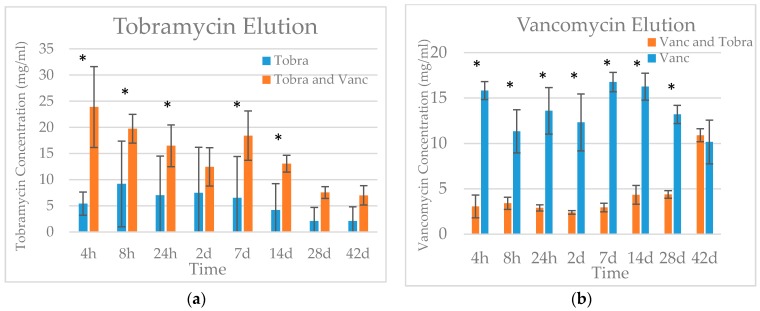
Tobramycin (**a**) and vancomycin (**b**) elution from calcium bone void filler (BVF) over 42 days. Mean ± standard deviation. * Signifies a statistical difference between the single and dual antibiotic loaded beads (*p* < 0.05).

Both tobramycin and vancomycin concentration remained above 1 mg/mL throughout the 42-day study (Figure 1). Tobramycin concentration was significantly higher in the tobramycin and vancomycin combination group than the tobramycin only group at time points 4 h, 8 h, 24 h, 7 d, and 14 d (*p* < 0.05). Conversely, vancomycin concentration was higher for the vancomycin-only group than the vancomycin and tobramycin combination group at all time points except day 42 (*p* < 0.001). 

### 2.3. Dissolution

**Figure 2:**
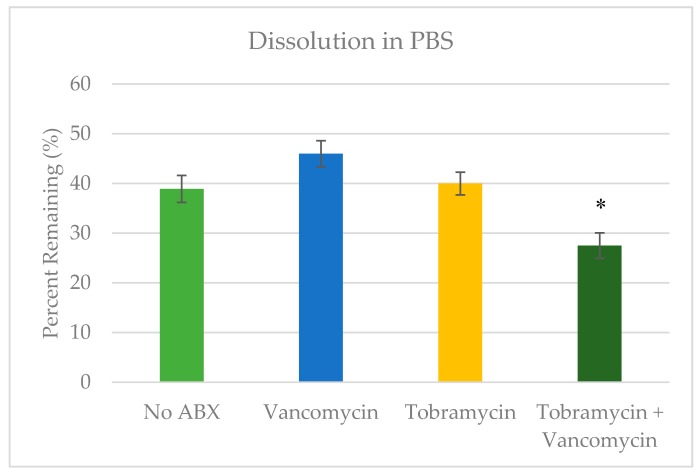
Calcium bone void filler (BVF) dissolution after 14 days in phosphate buffered saline (PBS). Mean ± standard deviation. * Signifies a statistical difference compared to the control (*p* < 0.05).

Beads containing BVF without antibiotics were reduced to 39% of the original mass after 14 days in phosphate buffered saline (PBS) (Figure 2). Beads containing tobramycin or vancomycin alone showed similar dissolution rates to the no antibiotic control; however, the tobramycin and vancomycin combination showed a higher rate of dissolution than non-antibiotic controls with only 27.5% mass remaining (*p =* 0.02). 

## 3. Discussion

Calcium sulfate, tricalcium phosphate, and hydroxyapatite have been used independently with success; however, each has disadvantages regarding dissolution and mechanical properties. Calcium sulfate has been used since the late eighteenth century to fill bony defects; however, it typically resorbs within 6–12 weeks before bone has fully filled the defect and matured [20,21]. Reports of increased inflammation, serous drainage, and questionable efficacy in vivo, likely due to the rapid resorption rate, have limited the use of pure calcium sulfate as a bone void filler [22,23,24,25]. Tricalcium phosphate (TCP)-based cements have a longer dissolution time, typically 6 months to 2 years, and have initial mechanical compression strength similar to that of cancellous bone [26,27]. TCP materials are better suited to filling large, load-bearing defects, but studies have shown that large defects still show a drop in mechanical properties at approximately 12 weeks when BVF dissolution has outpaced bone ingrowth [27]. Hydroxyapatite-based cements typically exhibit higher compression strength than TCP but take even longer to resorb, with some BVF degrading after 2–5 years and some highly crystalline scaffolds remaining permanently in the body [7,13,26,28]. These three BVF materials can form composites to achieve desired mechanical and biological properties while limiting disadvantages. Antibiotics mixed with calcium BVF reside within pores between the interlocking crystals of the calcium phosphate or calcium sulfate matrix and are released through either diffusion or BVF dissolution [19]. Drug elution from calcium phosphate-based BVF is typically described as diffusion-based, since elution outpaces dissolution; however, dissolution may result in increased porosity that improves elution at later time points [19]. When combining calcium sulfate and calcium phosphate BVF, the calcium sulfate will resorb first and create channels or pores throughout the structure [15]. It is therefore necessary to evaluate composite BVF elution properties separately from homogeneous calcium sulfate or calcium phosphate. Results of the current study show that the addition of vancomycin had little effect on BVF set time; however, tobramycin significantly prolonged the setting reaction. Clinically, the combination of vancomycin and tobramycin released from a local drug delivery matrix may be advantageous due to reported synergistic effects of these two antibiotics, particularly against a broad spectrum of microorganisms [29,30]. Triphasic BVF beads continued to elute for up to 42 days, more than the 4–6 weeks recommended for the treatment of most osteomyelitis cases [31]. 

Length of set time and preparation steps is a factor for consideration for surgeons choosing to include antibiotics in triphasic BVF. Set time for plain BVF and antibiotic BVF determined using a Vicat needle and by an orthopedic surgeon using tactile feedback. Use of ASTM standardized methods provides data that can easily be compared to prior studies, while qualitative assessment by an orthopedic surgeon can corroborate set time results using tactile feedback relevant to surgical conditions. The extended set time of triphasic BVF and tobramycin-loaded groups may be due to the documented hygroscopic nature of tobramycin [32]. There is a need for future studies to evaluate direct effects of the ambient humidity and temperature levels on set time. In a study by McLaren et al., it was suggested that generic tobramycin particles occupy more volume than proprietary formulations, which could hinder their crystallization [33]. The addition of vancomycin, however, had a minimal impact on set time. Due to the lengthy set time of BVF combined with 1.2 g tobramycin, future work will include studies with lower tobramycin concentrations, a smaller volume of deionized water in the BVF mixture, and various mixing techniques to reduce set time. 

Elution properties were assessed using a 42-day partial refreshment elution study. The BVF mass-to-fluid-volume ratio is based on prior studies that suggest that up to 40 calcium sulfate beads are packed into wounds with a total volume of 100 cc [34]. This was scaled back to an approximate BVF volume of 4 cc, which is comparable to the amount used in the treatment of osteomyelitis [35,36]. A partial refreshment elution study was performed to better approximate in vivo elution, which is believed to be slower than in full refreshment in vitro studies [34,37]. Concentrations of antibiotics eluted from triphasic BVF in this study suggest therapeutic efficacy over the course of bone regeneration. A similar partial refreshment elution study using calcium sulfate BVF showed that vancomycin and tobramycin concentrations dropped to less than one tenth of their peak concentration by day 42 [37]. In contrast, the vancomycin and tobramycin concentrations in this study remained at 61% and 21% of their respective peak concentrations at day 42, suggesting prolonged elution at later time points compared to plain calcium sulfate. Other studies with calcium sulfate have shown that vancomycin elution is more consistent and prolonged than that of tobramycin, as was seen in this study [38]. The reduced vancomycin elution from beads containing a combination of antibiotics is in contrast to prior research showing that vancomycin and tobramycin release from calcium sulfate was higher for combination groups than groups containing either antibiotic alone [39]. The HA or TCP components of triphasic BVF may have altered physiochemical interactions with vancomycin in the presence of tobramycin. The glycopeptide antibiotic vancomycin may show similar binding to mineral components of calcium-based BVF materials as proteins in the TGF-beta family [40,41]. The increased vancomycin elution from the combination group at day 42 also corresponds to the point at which tobramycin elution slows, suggesting that these composite beads are preferentially eluting tobramycin over vancomycin, although further work is needed to test this finding. Together these results suggest that the triphasic mixture has an extended antibiotic release profile compared to calcium sulfate [39,42]. The difference in loading concentrations between antibiotics limits our ability to make comparisons between antibiotics, although general profile comparisons can be made. These concentrations were selected to mimic the amounts and form of antibiotics available to surgeons in the operating room, thereby providing clinically relevant data. Partial media refreshment has been shown to cause slower elution rates than full refreshment studies, making it difficult to directly compare results of this study with others [34,37,42]. Although our results describe the behavior of triphasic BVF impregnated with antibiotics in vitro, the complex in vivo environment introduces many variables that may alter elution and/or dissolution [42]. Immune cells and osteoclasts, known to degrade calcium phosphate cements in vivo [43,44], may increase the rates reported in this study. 

A 14-day in vitro dissolution study was used, which compared the effects of antibiotics incorporation on dissolution in PBS. The 14-day time point was selected based on preliminary studies that suggested some of the beads may completely dissolve at longer time points. Dissolution was not affected by the addition of a single antibiotic. The BVF loaded with both antibiotics did show a significant increase in mass loss over 14 days in PBS, however. Prior studies with calcium sulfate have shown that the addition of antibiotics can affect the crystallinity of the BVF [33]. It is therefore possible that the increased dissolution rate seen in the combination group is due to reduced crystallinity of the triphasic BVF, resulting in quicker dissolution of the calcium sulfate. Future studies should include x-ray crystallography and/or mechanical testing of combination group to test this hypothesis.

In conclusion, the addition of antibiotics to Osteoboost BVF affected set time depending on the type of antibiotic used. Using combinations of antibiotics could be beneficial in using synergistic effects of aminoglycosides and vancomycin to prevent osteomyelitis. This study aimed to guide surgeons on what to expect when using vancomycin and tobramycin with triphasic BVF. Future studies will focus on additional antimicrobials and antifungal agents. 

## 4. Materials and Methods 

### 4.1. Bead Preparation

Antibiotics used in the BVF mixture in this study included tobramycin sulfate (Research Products International, Prospect, IL, USA) and vancomycin hydrochloride (MP Biomedicals, Solon, OH, USA), or a combination of both). Osteoboost^®^ triphasic BVF beads (OsteoRemedies, Memphis, TN, USA) were prepared according to manufacturer instructions by mixing BVF powder with the appropriate antibiotic powder and 7 mL of deionized water for 60 s, packing the paste into rubber molds with a spatula, and waiting until the beads hardened. A Vicat needle was used to determine set time according to ASTM C472. Briefly, the BVF powder was mixed with the appropriate antibiotic and quantity of deionized water and placed in a cylindrical mold. The Vicat needle was set to the top edge of the mixture and released. Set time was determined when the needle failed to penetrate half the depth of the BVF. Separately, the BVF was mixed by an orthopedic surgeon and packed into rubber molds provided with the BVF. The surgeon qualitatively declared set time based on the hardness of the BVF upon probing with a plastic spatula. Bead groups included powdered vancomycin (1 g), powdered tobramycin (1.2 g), or both vancomycin and tobramycin (1 g and 1.2 g, respectively). Each group consisted of 4 samples (*n* = 4), and the beads were mixed by an orthopedic surgeon in similar conditions to that of an operation room. 

### 4.2. Elution

A 42-day elution study was carried out to determine the concentrations of antibiotics released from the BVF pellets. From each group, 3 g of pellets were submersed in 4 mL of phosphate buffered saline (PBS) at 37 °C with shaking (*n* = 4). Elution samples were collected at 4 h, 8 h, 1 day, 2 days, 7 days, 14 days, 28 days, and 42 days. At each time point, 1.5 mL elution media was removed and replaced with fresh PBS. Antibiotic concentration was then determined using high performance liquid chromatography (Dionex Ultimate 3000, Thermo Fisher Scientific, Waltham, MA, USA) with a C18 column (Hypersil Gold, Thermo Fisher Scientific, Waltham, MA, USA). A pre-column amine derivatization method was used to enable fluorescence detection of tobramycin [45]. 

### 4.3. Dissolution

Dissolution was determined for all antibiotic groups and 1 control group without antibiotics (*n* = 4). One pre-weighed pellet per group was placed in 4 mL PBS that was refreshed every two days for a duration of 14 days. On day 14, samples were dried in a vacuum oven, and percent mass loss was calculated. 

### 4.4. Statistical Analysis

All statistical tests were performed using SigmaPlot software (Systat Software, Inc., San Jose, CA, USA). Two-way analysis of variance (ANOVA) with Holm-Sidak post-hoc was used to test for differences in antibiotic elution rate between groups. One-way ANOVA with Holm-Sidak post-hoc was used to test for differences in dissolution rate. A significance level of 0.05 was used for determining statistical significance between groups.
